# Increasing smoking intensity is associated with increased disease activity in axial spondyloarthritis

**DOI:** 10.1007/s00296-016-3590-4

**Published:** 2016-11-04

**Authors:** Sizheng Zhao, Benjamin Challoner, Mohammed Khattak, Robert J. Moots, Nicola J. Goodson

**Affiliations:** 1 Academic Rheumatology Department, Clinical Sciences Centre, Aintree University Hospital, Liverpool, UK; 2 Institute of Ageing and Chronic Disease, University of Liverpool, Liverpool, UK

**Keywords:** Axial spondyloarthritis, Ankylosing spondylitis, Cigarette smoking, Disease activity, Functional impairment

## Abstract

A history of ever-smoking appears to be associated with a more severe disease phenotype in axial spondyloarthritis (axSpA). However, evidence is sparse for the effect of increased smoking exposure on disease outcomes or whether smoking reduction or cessation improves outcomes. The aim of this study was to explore whether a dose–response relationship exists between pack-years and disease activity and functional impairment in axSpA. Consecutive patients meeting ASAS criteria for axial SpA were recruited from a spondyloarthritis service. The associations between pack-years of smoking and: (1) disease activity (BASDAI/ASDAS), (2) spinal pain, (3) functional impairment (BASFI) and (4) inflammatory markers were explored using multivariable linear models, adjusted for age, gender and use of TNF inhibition (TNFi) therapy. Pack-years were categorised into four groups (<10, 11–20, 21–40, >40) and analysed with light smoking (<10) as reference. Two hundred and thirty-eight axSpA patients were recruited: 76% were male, mean age 46.4 years (SD ± 13.7), and 33% were treated with TNFi. One hundred and twelve patients reported history of ever-smoking with median pack-year 20 [IQR10-30]. Compared to light smokers, those with higher categories of smoking exposures had higher BASDAI (21–40 pack-years, *β* = 1.6 (95% CI 0.28, 2.95); >40, *β* = 2.6 (0.54, 3.56)), higher BASFI (21–40, *β* = 2.1 (0.42, 4.80); >40, *β* = 3.2 (0.76, 5.71)), and higher ASDAS (21–40, *β* = 0.82 (0.14, 1.51)). This cross-sectional study demonstrated that smoking is associated with increased axSpA severity markers in a dose–response manner. Particular effort should be made to restrict smoking exposure early before accruing a significant number of pack-years.

## Introduction

Axial spondyloarthritis (axSpA) is a chronic inflammatory disease involving the entheses. Musculoskeletal involvement is predominantly axial (sacroiliitis, spondylitis) leading to inflammatory back pain, but can be also involve peripheral joints and tendons (arthritis, enthesitis, dactylitis). AxSpA can be further divided into ‘radiographic’ (ankylosing spondylitis, AS) and ‘non-radiographic’ (nr-axSpA), depending on whether definitive structural changes are evident on plain radiographs of sacroiliac joints [1, 2]. Patients with nr-axSpA may display active inflammation on magnetic resonance imaging (MRI). Nr-axSpA may represent early AS but may also be a limited form that is symptomatically similar but does not lead to structural changes. Since delineation of nr-axSpA and AS is artificial and unreliable, both forms have been suggested to be one disease [3].

Cigarette smoking appears to have an important role in axSpA. Studies have demonstrated that smoking is associated with incident AS [4] and earlier onset of axSpA [5]. Smokers with AS have increased disease activity, functional impairment and reduced quality of life [6, 7]. The reasons for these associations are not well understood but may reflect increased inflammatory burden associated with smoking or possibly confounding effects of socioeconomic class. However, previous studies exploring association between smoking and disease severity have utilised cross-sectional study designs, which limit causal inference. Exploring the dose–response relationship between smoking and disease severity may provide more evidence for causal relationship. Existing studies have mostly been in small cohorts with varying results and many methodological limitations [8–13]. The aims of this study were to test the hypothesis that pack-years of cumulative exposure to cigarette smoking in ever-smokers are associated with disease activity and functional impairment in axSpA and to quantify its effect size.

## Method

Consecutive patients attending a tertiary referral spondyloarthritis service, in a UK hospital setting, were recruited between September 2010 and December 2015. Patients were included if they fulfilled the ASAS criteria for axial SpA [2].

Clinical data were collected during routine out-patient assessments. Patient characteristics were recorded including: age, gender, body mass index (BMI). Smoking status was categorised as current smoker, ex-smoker and non-smoker. For patients who have ever smoked (current and ex-smokers), total cumulative exposure was estimated with pack-years: one pack-year equals cigarettes per day multiplied by number of years smoked divided by 20. In addition, disease variables were recorded including: classification as AS, symptom duration, duration since diagnosis, HLA-B27 status if available and extra-axial disease features (peripheral joint involvement, psoriasis, uveitis and inflammatory bowel disease). HLA-B27 was not systematically tested in this real-life cohort and therefore was missing in a significant proportion. Current use of non-steroidal anti-inflammatory drugs (NSAIDs) and tumour necrosis factor inhibition (TNFi) was also recorded. Disease activity and functional status were assessed using numeric rating scale versions of the Bath AS Disease Activity Index (BASDAI), spinal pain (spNRS) and Bath AS Functional Index (BASFI). Patient global was introduced into clinical practice after study commencement and was therefore incomplete with data missing at random. Blood samples were taken on the day of assessment, for analysis of erythrocyte sedimentation rate (ESR) and C-reactive protein (CRP). Ankylosing Spondylitis Disease Activity Score (ASDAS) was calculated using CRP for patients with complete data [14].

Statistical analyses were performed using Stata12. Comparison of patient and disease characteristics between the three smoking categories were performed using ANOVA for Gaussian, Kruskal–Wallis test (or Mann–Whitney *U* if two categories) for non-Gaussian and Chi-squared or Fisher’s exact test for categorical variables. Multivariable linear regression models were used to explore the association between each measure of disease activity (BASDAI, ASDAS, spNRS, BASFI, CRP and ESR) in turn as the dependent variable, and non-/ever-smoking as the dichotomous independent variable, adjusted for age, gender and use of TNFi. Symptom duration was not included as a covariate, given its collinearity with age. Due to their non-Gaussian distribution, ESR and CRP were log-transformed prior to regression (ln(ESR), ln(CRP)). ASDAS was regressed using complete case analysis and again using multiple imputation for those with missing patient global scores. Multiple imputation was performed using multivariate normal distribution with 30 imputed datasets. Variables used in the imputation model were those specified in the regression models, with BASDAI in addition as an auxiliary variable [15]. The aim of this study was to explore associations between smoking and disease activity. As patients with both AS and nr-axSpA can manifest similar levels of disease activity [3], these patients were grouped together in the analyses.

To explore the effect of pack-years on the above markers of disease severity, the same multivariable linear models were used, with pack-years as the independent variable categorised into four groups (<10, 11–20, 21–40 and >40 pack-years) and analysed as dummy variables with <10 pack-years as the reference. Categorisation was arbitrary to give similar groups sizes. Results were presented as coefficients and 95% confidence intervals (95% CI). Residuals from each model were tested against normal distribution using Shapiro–Francia test.

This study received UK Research Ethics Committee approval (15/LO/1519).

## Results

The study recruited 238 patients with established axSpA. The cohort was predominantly male (76%) with mean age of 46.4 years (SD ± 13.7), median symptom duration of 17.1 years [inter-quartile rage (IQR) 8.4, 29.3] and median duration since diagnosis of 5.0 years [IQR 0.8, 14.8]. HLA-B27 was measured in 61% of the cohort and of these 61% were positive. AS was present in 83%. Use of NSAIDs was reported by 163 (68%) patients, and 79 (33%) were treated with TNFi.

At the time of assessment, a history of ever-smoking was reported by 112 (47%) patients with 78 (33%) reporting current smoking. Among ever-smokers, the median pack-year was 20 [IQR 10, 30]. The mean age of ex-smokers was older at 52 years, with similar ages of current smokers at 45 years and non-smokers at 46 years. Similarly, symptom duration was longest in the ex-smoker group at 23 years. There were also more males (91%) in the ex-smoking group than other groups. The median BASDAI was 5.7 [IQR 3.3, 7.6] and BASFI 5.7 [3.3, 7.6]. ASDAS was available for 188 (79%) patients with mean of 2.7 (SD ± 1.14). Patient demographics and disease characteristics compared between smoking categories are shown in Table 1.

**Table 1:** Patient and disease characteristics of the cohort and for each smoking category

	All	Non-smokers	Ex-smokers	Current smokers	*P* value
Number of patients	238	126 (52.9%)	34 (14.3%)	78 (32.8%)	
Age	46.4 (13.8)	46.1 (14.2)	51.7 (12.1)	44.6 (13.2)	0.046
Male gender	180 (75.6%)	87 (69.1%)	31 (91.2%)	62 (79.5%)	0.018
BMI^a^	27.6 [25.1, 31.0]	27.4 [25.2, 30.5]	29.0 [25.8, 32.6]	27.6 [24.7, 31.0]	0.443
AS diagnosis	197 (82.8%)	99 (78.6%)	27 (79.4%)	71 (91.0%)	0.062
HLA-B27 status^a^	89 (61.0%)	49 (59.8%)	14 (70.0%)	26 (59.1%)	0.670
Symptom duration (years)	16.5 [8.4, 28.4]	15.0 [6.4, 30.0]	23.1 [12.6, 33.1]	16.3 [8.8. 26.7]	0.033
Diagnosis duration (years)	5.0 [0.8, 14.8]	4.7 [0.8, 15.2]	4.0 [1.2, 14.8]	0.9 [5.4, 14.4]	0.802
Pack-years	0 [0, 15]	0	20 [10, 30]	15 [9, 30]	0.151^b^
BASDAI	5.7 [3.3, 7.6]	5.2 [3.0, 7.5]	6.0 [4.1, 8.2]	6.5 [3.6, 7.6]	0.132
Spinal pain	6.0 [3.0, 8.0]	5.0 [2.0, 8.0]	7.0 [3.0, 8.0]	7.0 [3.0, 8.0]	0.254
ASDAS^a^	2.70 (1.14)	2.39 (1.16)	3.28 (1.08)	2.96 (0.98)	0.001
BASFI	5.7 [2.9, 7.6]	5.0 [2.3, 7.4]	6.9 [3.1, 8.1]	5.9 [3.2, 7.7]	0.116
CRP (mg/l)^a^	3 [1, 9]	3 [1, 8]	5 [2, 11]	5 [1, 11]	0.131
ESR (mm/hr) ^a^	8 [5, 21]	8 [5, 19]	10 [6, 26]	10[5, 26]	0.123
Peripheral joint involvement	55 (23.6%)	35 (28.0%)	8 (23.5%)	12 (16.2%)	0.167
Psoriasis	39 (16.4%)	21 (16.70%)	6 (17.7%)	12 (15.4%)	0.949
Uveitis	64 (26.9%)	38 (30.2%)	12 (35.3%)	14 (18.0%)	0.079
IBD	22 (9.2%)	14 (11.1%)	3 (8.8%)	5 (6.4%)	0.576
TNFi	79 (33.2%)	39 (31.0%)	12 (35.3%)	28 (35.9%)	0.737
NSAID	163 (68.5%)	87 (69.1%)	20 (58.8%)	56 (71.8%)	0.390

Data are presented in *n* (%), mean (SD), median [IQR] and comparison used Chi-squared/Fisher’s exact, ANOVA and Kruskal–Wallis tests, respectively

*axSpA* axial spondyloarthritis, *ASDAS* Ankylosing Spondylitis Disease Activity Score, *BMI* body mass index, *IBD* inflammatory bowel disease, *TNFi* TNF inhibition therapy, *NSAID* non-steroidal anti-inflammatory drugs

^a^ BMI complete data in 190; HLAB27 status known in 146; ASDAS in 188; CRP in 231; ESR in 230

^b^ Mann–Whitney *U* test

No significant differences were seen between smoking groups for duration since diagnosis. Proportion of AS was higher in current smokers compared with rest of the cohort (91 vs. 79%, *P* = 0.019).

Extra-axial features were similarly prevalent between the three smoking categories. However, the prevalence of uveitis was lower in current smokers compared to rest of the cohort (18 vs. 31%, *P* = 0.03).

Median disease severity measures were all higher in current and ex-smokers than non-smokers, but there were no statistically significant differences between the three smoking categories. Compared to non-smokers, ever-smokers had significantly higher BASDAI, ASDAS and BASFI, but not spNRS, ESR or CRP (data not shown). The use of TNFi and NSAIDs was similar between three groups.

In all multivariable models, no significant interactions were found between independent variables. NSAID-use and HLA-B27 were not included as covariates as they did not demonstrate significant association in, or improvement to, the models. In multivariable linear regression models, ever-smoking was independently associated with higher BASDAI (*β* = 0.91, 95% CI 0.26, 1.55), spNRS (*β* = 0.85, 95% CI 0.11, 1.59) and BASFI (*β* = 0.82, 95% CI 0.10, 1.53). Ever-smoking was also associated with ASDAS using complete case analysis (*β* = 0.70, 95% CI 0.39, 1.01) and multiply imputed data (*β* = 0.71, 95% CI 0.40, 1.01). However, ever-smoking was not associated with CRP (*β* = 2.68, 95% CI −1.85, 7.21) or ESR (*β* = 4.34, 95% CI −0.91, 9.58).

Multivariable linear models exploring associations with pack-year categories demonstrated significant dose-related increase for BASDAI, ASDAS and BASFI but not spNRS, CRP or ESR (Table 2). Compared to the <10 pack-year reference group, smokers with 11–20 pack-years did not have significantly higher BASDAI or BASFI. However, heavier smokers with 21–40 pack-years had scores 1.6 higher for BASDAI and 2.1 higher for BASFI than the reference group. The group with >40 pack-year exposure had scores 2.6 and 3.2 higher than the reference group for BASDAI and BASFI, respectively. Pack-years were also associated with ASDAS; however, this was not significant in the highest smoking category. Smokers with 21–40 pack-years had nearly one point higher ASDAS than the reference group. Residuals from all regression models were not statistically different from the normal distribution.

**Table 2:** Multivariable linear regression models of association between pack-year categories and measures of disease activity (adjusted for age, gender and use of TNFi)

	<10 pack-years *n* = 40	11–20 pack-years *n* = 34	21–40 pack-years *n* = 32	>40 pack-years *n* = 6
BASDAI	Reference	0.55 (−0.55, 1.65)	**1.62 (0.28, 2.95)**	**2.61 (0.42, 4.80)**
ASDAS complete case analysis	Reference	0.31 (−0.23, 0.84)	**0.82 (0.14, 1.51)**	0.54 (−0.53, 1.62)
ASDAS multiply imputed	Reference	0.33 (−0.20, 0.86)	**0.92 (0.25, 1.59)**	0.88 (−17, 1.93)
Spinal pain	Reference	0.54 (−0.77, 1.85)	1.34 (−0.25, 2.93)	1.40 (−1.21, 4.01)
BASFI	Reference	0.95 (−0.29, 2.20)	**2.05 (0.54, 3.56)**	**3.24 (0.76, 5.71)**
CRP	Reference	0.08 (−0.56, 0.72)	0.53 (−0.24, 1.30)	1.08 (−0.22, 2.37)
ESR	Reference	0.27 (−0.26, 0.80)	0.28 (−0.38, 0.93)	0.71 (−0.33, 1.76)

Statistically significant values are represented in bold

Results are presented as *β* coefficient (95% confidence interval)

*BASDAI* Bath Ankylosing Spondylitis Disease Activity Index, *ASDAS* Ankylosing Spondylitis Disease Activity Score, *BASFI* Bath AS Functional Index, *CRP* C-reactive protein, *ESR* erythrocyte sedimentation rate

## Discussion

This cross-sectional study of an axSpA cohort has demonstrated that smoking is associated with increased disease activity and functional impairment. In axSpA participants reporting history of ever-smoking, increased cumulative smoking exposure was observed to have a clear independent and significant association with BASDAI and BASFI. This was irrespective of whether participants were current or ex-smokers. Compared to light smoking exposures (<10 pack-years), those with higher pack-year smoking histories had increased BASDAI and BASFI. Ever-smokers with 21–40 pack-year history had BASDAI and BASFI approximately two points higher than light smokers. Scores were highest in those with more than 40 pack-years of smoking.

A trend was also demonstrated for the more sensitive index ASDAS. The loss of statistical significance for the heaviest pack-year category is likely due to the small size of this group. No significant associations were demonstrated for ESR or CRP. The effect sizes increased with increasing pack-year categories; therefore, it may be that statistical power was insufficient. CRP and ESR are not specific markers of axSpA disease activity and may be influenced by other factors.

These findings are in agreement with three of the six existing studies of pack-years in AS [7, 8, 13]. Chen et al. [8] reported unadjusted correlation between pack-years and BASFI, but not BASDAI, ESR or CRP. An Iranian study found pack-years to be associated with BASDAI and of quality of life (QoL) but not BASMI in cohort of very light current smokers (median of 0.6 pack-years) [13]. The largest and most robust study found associations between categories of pack-years and all of their markers including BASDAI, BASFI, pain numerical rating score and two measures of QoL [7].

The three remaining studies reported no associations between markers of disease severity and pack-years [11, 12, 16]. There are several possible explanations for the inconsistent results. Firstly, it is important to consider confounders when studying smoking and disease activity. Some studies used simple correlation [8, 16] and others used variable approaches when selecting covariates [12, 13]. Secondly, most studies recruited smaller cohorts with fewer (35–84 patients) entered into pack-year analysis [8, 11–13]. The larger studies were also not without limitations: one was survey-based with 64% response rate to postal survey and no adjustment for TNFi [7] and the other explored association with BASFI alone with unadjusted correlation [16]. Our study is the first to quantify effect size of the association between pack-years and markers of disease activity using multi-adjusted analysis.

Our finding that only heavier smokers (>20 pack-years) had significantly higher disease activity was also observed by Mattey et al. [7] In their survey study of AS patients using a test for trend, BASDAI, BASFI, pain and two QoL measures were all associated with higher smoking exposure (>15 pack-years), but not for those with ≤15 pack-years. This is more likely as a result of statistical rather than biological effects.

In our axSpA cohort, those with a history of ever-smoking had more severe disease than non-smoking participants. This was in agreement with previous studies [5, 7, 8, 11]. Ex-smokers were older and had longer symptom duration than current or non-smokers. This was also observed by Mattey et al. and may be that smoking tends to start at a younger age.

AS was more prevalent in current than non-current smokers. This could not be explained by age or gender (factors associated with radiographic progression); current smokers were younger with shorter disease and symptom duration and did not have more males than other groups. Other studies have reported greater radiographic progression in AS smokers [9, 10, 12].

It was also interesting to observe that uveitis was reported less frequently in current smokers, compared to ex-/non-smokers. Cigarette smoking is a recognised risk factor for uveitis in the general population [17]. However, development of the extra-articular disease manifestation may trigger smoking cessation behaviour. This finding was also reported in a larger Scottish cohort, where the authors hypothesised that cigarette smoke may be an irritant in those with uveitis leading to increased rates of cessation, or that the effect of uveitis on quality of life may have a particular impact on health behaviour [18]. This may also be one of many risk factor paradoxes due to collider-stratification bias described in detail in 19.

The disparity between symptom and disease duration in this cohort suggests a prolonged delay to referral to the SpA service for diagnosis. This reflects the ongoing need to raise awareness of axSpA in primary care. Continuous NSAIDs was not promoted at this spondyloarthritis service given side effects and uncertain benefit [20]. Patients in this cohort generally took NSAIDs on-demand, and NSAID index was therefore not used.

There were several limitations to this study. The cross-sectional design limits ability to infer causality between smoking and disease severity. However, the clear dose–response relationship provides some support for smoking causing more severe disease phenotype in axSpA. Studies of smoking status are always subject to social desirability bias. It is possible that patients are misclassified by self-reported smoking status. This is especially true for ex- and current-smoking groups and is one reason that they were analysed together under ever-smokers. Future studies should set more stringent definitions of ex-smoking. This will allow separately analysis of dose–response relationship in ex- and current smokers. The categorisation of pack-years left only a small subgroup with heaviest (>40) pack-year exposure. Importantly, due to limited data collection, no adjustment was made for socioeconomic class in this study, although pack-years have been associated with disease activity independent of socioeconomic class [7, 18].

In conclusion, this study found that cigarette smoking was associated with increased axSpA severity in a clear dose–response manner. This dose–response relationship persists even in those who have quit smoking. Therefore, smoking cessation may be more beneficial if implemented before accruing a large number of pack-years. It is important for clinicians to help patients with smoking cessation and smoking reduction as early as possible.

## References

[1] van der Linden S, Valkenburg HA, Cats A (1984). Evaluation of diagnostic criteria for ankylosing spondylitis. A proposal for modification of the New York criteria. Arthritis Rheum.

[2] Rudwaleit M, Jurik AG, Hermann KG, Landewe R, van der Heijde D, Baraliakos X, Marzo-Ortega H, Ostergaard M, Braun J, Sieper J (2009). Defining active sacroiliitis on magnetic resonance imaging (MRI) for classification of axial spondyloarthritis: a consensual approach by the ASAS/OMERACT MRI group. Ann Rheum Dis.

[3] Baraliakos X, Braun J (2015). Non-radiographic axial spondyloarthritis and ankylosing spondylitis: what are the similarities and differences?. RMD Open.

[4] Videm V, Cortes A, Thomas R, Brown MA (2014). Current smoking is associated with incident ankylosing spondylitis—the HUNT population-based Norwegian health study. J Rheumatol.

[5] Chung HY, Machado P, van der Heijde D, D’Agostino MA, Dougados M (2012). Smokers in early axial spondyloarthritis have earlier disease onset, more disease activity, inflammation and damage, and poorer function and health-related quality of life: results from the DESIR cohort. Ann Rheum Dis.

[6] Wendling D, Prati C (2013). Spondyloarthritis and smoking: towards a new insight into the disease. Expert Rev Clin Immunol.

[7] Mattey DL, Dawson SR, Healey EL, Packham JC (2011). Relationship between smoking and patient-reported measures of disease outcome in ankylosing spondylitis. J Rheumatol.

[8] Chen CH, Chen HA, Lu CL, Liao HT, Liu CH, Tsai CY, Chou CT (2013). Association of cigarette smoking with Chinese ankylosing spondylitis patients in Taiwan: a poor disease outcome in systemic inflammation, functional ability, and physical mobility. Clin Rheumatol.

[9] Haroon N, Inman RD, Learch TJ, Weisman MH, Lee M, Rahbar MH, Ward MM, Reveille JD, Gensler LS (2013). The impact of tumor necrosis factor alpha inhibitors on radiographic progression in ankylosing spondylitis. Arthritis Rheum.

[10] Klingberg E, Nurkkala M, Carlsten H, Forsblad-d’Elia H (2014). Biomarkers of bone metabolism in ankylosing spondylitis in relation to osteoproliferation and osteoporosis. J Rheumatol.

[11] Reed MD, Dharmage S, Boers A, Martin BJ, Buchanan RR, Schachna L (2008). Ankylosing spondylitis: an Australian experience. Intern Med J.

[12] Sakellariou GT, Anastasilakis AD, Kenanidis E, Potoupnis M, Tsiridis E, Savvidis M, Kartalis N, Sayegh FE (2015). The effect of smoking on clinical and radiographic variables, and acute phase reactants in patients with ankylosing spondylitis. Rheumatol Int.

[13] Fallahi S, Jamshidi AR, Gharibdoost F, Mahmoudi M, Ahmadzadeh N, Nicknam MH (2013). The correlation between pack-years of smoking and disease activity, quality of life, spinal mobility, and sacroiliitis grading in patients with ankylosing spondylitis. Turk J Rheumatol.

[14] Lukas C, Landewe R, Sieper J, Dougados M, Davis J, Braun J, van der Linden S, van der Heijde D, Assessment of SpondyloArthritis international S (2009). Development of an ASAS-endorsed disease activity score (ASDAS) in patients with ankylosing spondylitis. Ann Rheum Dis.

[15] Young R, Johnson D (2011) Imputing the missing Y’s: implications for survey producers and survey users. Proceedings of the AAPOR conference abstracts, pp 6242–6248

[16] Ward MM, Weisman MH, Davis JC, Reveille JD (2005). Risk factors for functional limitations in patients with long-standing ankylosing spondylitis. Arthritis Rheum.

[17] Roesel M, Ruttig A, Schumacher C, Heinz C, Heiligenhaus A (2011). Smoking complicates the course of non-infectious uveitis. Graefes Arch Clin Exp Ophthalmol.

[18] Jones GT, Ratz T, Dean LE, Macfarlane GJ, Atzeni F (2014). The effect of smoking cessation in ankylosing spondylitis—results from the Scotland Registry for Ankylosing Spondylitis (SIRAS). Clin Exp Rheumatol.

[19] Choi HK, Nguyen US, Niu J, Danaei G, Zhang Y (2014). Selection bias in rheumatic disease research. Nat Rev Rheumatol.

[20] Kroon FP, van der Burg LR, Ramiro S, Landewe RB, Buchbinder R, Falzon L, van der Heijde D (2015). Non-steroidal anti-inflammatory drugs (NSAIDs) for axial spondyloarthritis (ankylosing spondylitis and non-radiographic axial spondyloarthritis). Cochrane Database Syst Rev.

